# Supplementary material for: Association of metabolically healthy obesity and elevated risk of coronary artery calcification: a systematic review and meta-analysis
Source: PeerJ. 2020 Mar 26;8:e8815. doi: 10.7717/peerj.8815 (PMC7103199; doi:10.7717/peerj.8815)
Supplement: Table S1 — From: Moher D, Liberati A, Tetzlaff J, Altman DG, The PRISMA Group (2009). Preferred Reporting Items for Systematic Reviews and Meta-Analyses: The PRISMA Statement. PLoS Med 6(7): e1000097. doi:10.1371/journal.pmed1000097 For more information, visit: www.prisma-statement.org . [file peerj-08-8815-s002.docx]

**Supplemental Table S1. PRISMA Checklist for Meta-analyses of Observational Studies**

| **Section/topic** | **#** | | **Checklist item** | **Reported on page** | **Text excerpted from the manuscript** |  |
| --- | --- | --- | --- | --- | --- | --- |
| **TITLE** | | | |  |  |  |
| Title | 1 | | Identify the report as a systematic review, meta-analysis, or both. | 2-3 | Association of Metabolically Healthy Obesity and Elevated. Risk of Coronary Artery Calcification: A Systematic Review and Meta-analysis |  |
| **ABSTRACT** | | | |  |  |  |
| Structured summary | 2 | | Provide a structured summary including, as applicable: background; objectives; data sources; study eligibility criteria, participants, and interventions; study appraisal and synthesis methods; results; limitations; conclusions and implications of key findings; systematic review registration number. | 2-3 | Our abstract Included background; objectives; data sources; study eligibility criteria, participants, and interventions; study appraisal and synthesis methods; results; limitations; conclusions and implications of key findings; systematic review registration number. |  |
| **INTRODUCTION** | | | |  |  |  |
| Rationale | 3 | | Describe the rationale for the review in the context of what is already known. | 4-5 | Obesity has become a major global health burden. Some studies report that MHO individuals show similar risk of cardiovascular disease (CVD) compared with metabolically healthy non-obese (MHNO) individuals. It remains conflict. |  |
| Objectives | 4 | | Provide an explicit statement of questions being addressed with reference to participants, interventions, comparisons, outcomes, and study design (PICOS). | 5-6 | The objective of this study was to systematically and comprehensively explore the relationship between MHO and CAC risk in comparison to MHNO participants. |  |
|  |  | |  |  |  |  |
| **METHODS** | | | |  |  |  |
| Protocol and registration | 5 | | Indicate if a review protocol exists, if and where it can be accessed (e.g., Web address), and, if available, provide registration information including registration number. | 7 | Following a pre-established protocol registered on PROSPERO (CRD 42019135006) |  |
| **Section/topic** | **#** | | **Checklist item** | **Reported on page** | **Text excerpted from the manuscript** |  |
| Eligibility criteria | 6 | | Specify study characteristics (e.g., PICOS, length of follow-up) and report characteristics (e.g., years considered, language, publication status) used as criteria for eligibility, giving rationale. | 7-8 | Articles were selected if the title or abstract indicated that the study analyzed the association between CAC and MHO. Parameter ranges for language, year of publication, article type, and participant characteristics including age were not limited to enable a relatively comprehensive search. |  |
| Information sources | 7 | | Describe all information sources (e.g., databases with dates of coverage, contact with study authors to identify additional studies) in the search and date last searched. | 7 | Using the Medline, EMBASE, and Cochrane library databases supplemented with the manual review of the reference list of obtained articles up to April 19, 2019. |  |
| Search | 8 | | Present full electronic search strategy for at least one database, including any limits used, such that it could be repeated. | 7 | Full search strategies are shown in Supplemental Table 2 |  |
| Study selection | 9 | | State the process for selecting studies (i.e., screening, eligibility, included in systematic review, and, if applicable, included in the meta-analysis). | 7-8 | We performed a systematic literature search. The searches  were conducted by two authors and any disagreements were  resolved via discussion. Then, we performed a manual search  of references from relevant publications, as well as previous  reviews and meta-analyses. |  |
| Data collection process | 10 | | Describe method of data extraction from reports (e.g., piloted forms, independently, in duplicate) and any processes for obtaining and confirming data from investigators. | 7-8 | We extracted the following information: first author’s name, year of publication, description of the study population (country, number of participants/cases per MHO phenotype, mean age, sex proportion, duration of follow-up in cohort studies), study design, definition of metabolically healthy and obese, diagnostic methods of CAC, adjusted variables, and main outcome presented with OR (95%CI) using MHNO as the reference. Some studies had dulplicate population. Included studies was assessed independently by two authors. |  |
| Data items | 11 | | List and define all variables for which data were sought (e.g., PICOS, funding sources) and any assumptions and simplifications made. | 8-9 | Extracted the following information: first author’s name, year of publication, description of the study population, study design, definition of metabolically healthy and obese, diagnostic methods of CAC, adjusted variables, and main outcome. |  |
| **Section/topic** | **#** | | **Checklist item** | **Reported on page** | **Text excerpted from the manuscript** |  |
| Risk of bias in individual studies | 12 | | Describe methods used for assessing risk of bias of individual studies (including specification of whether this was done at the study or outcome level), and how this information is to be used in any data synthesis. | 9 | Using the Newcastle Ottawa Scale (NOS) |  |
| Summary measures | 13 | | State the principal summary measures (e.g., risk ratio, difference in means). | 9 | We calculated pooled odds ratios (ORs) with 95% CIs for estimating the risk of CAC progression in MHO compared with MHNO. |  |
| Synthesis of results | 14 | | Describe the methods of handling data and combining results of studies, if done, including measures of consistency (e.g., I^2^) for each meta-analysis. | 9-10 | We employed a random effects model using DerSimonian and Laird’s method under an assumption of non-identical true effect sizes. |  |
| Risk of bias across studies | | 15 | Specify any assessment of risk of bias that may affect the cumulative evidence (e.g., publication bias, selective reporting within studies). | 9-10 | Publication bias was evaluated by funnel plots and Egger’s tests with a significant publication bias defined by a p value < 0.1. | |
| Additional analyses | | 16 | Describe methods of additional analyses (e.g., sensitivity or subgroup analyses, meta-regression), if done, indicating which were pre-specified. | 9-10 | Subgroup analysis, sensitivity analysis, meta-regression was described | |
| **RESULTS** | | | |  |  | |
| Study selection | | 17 | Give numbers of studies screened, assessed for eligibility, and included in the review, with reasons for exclusions at each stage, ideally with a flow diagram. | 11 | A total of 9 observational studies including cohort and cross-sectional studies were included in our systematic review.  (Figure 1) | |
| Study characteristics | | 18 | For each study, present characteristics for which data were extracted (e.g., study size, PICOS, follow-up period) and provide the citations. | 11-12 | The general demographic characteristics of subjects in the included studies are summarized in Table 1. | |
| **Section/topic** | **#** | | **Checklist item** | **Reported on page** | **Text excerpted from the manuscript** |  |
| Risk of bias within studies | | 19 | Present data on risk of bias of each study and, if available, any outcome level assessment (see item 12). | 13 | All cohort studies achieved at least eight out of nine stars on the NOS quality assessment scale. The cross-sectional studies scored from seven to nine out of ten stars, indicating that all included studies were of good quality. (Table S3) | |
| Results of individual studies | | 20 | For all outcomes considered (benefits or harms), present, for each study: (a) simple summary data for each intervention group (b) effect estimates and confidence intervals, ideally with a forest plot. | 13 | The results are shown in Figure 2 | |
| Synthesis of results | | 21 | Present results of each meta-analysis done, including confidence intervals and measures of consistency. | 13 | Participants with MHO had significantly higher risks of CAC than those with MHNO (OR = 1.38, 95% CI [1.12, 1.69], I2 = 44%) | |
| Risk of bias across studies | | 22 | Present results of any assessment of risk of bias across studies (see Item 15). | 14 | No significant publication bias was detected by Egger’s tests (p = 0.59) and there was no substantial asymmetry in the funnel plot (Figure. S4). | |
| Additional analysis | | 23 | Give results of additional analyses, if done (e.g., sensitivity or subgroup analyses, meta-regression [see Item 16]). | 13,14 | 1. Analyzing data in subgroups.  2. Performing meta-regression analyses for potential effect modifiers, consisting of sex, age, and smoking status.  3. Performing a sensitivity analysis by excluding one article in which the reference group was MHNW. | |
| **DISCUSSION** | | | |  |  | |
| Summary of evidence | | 24 | Summarize the main findings including the strength of evidence for each main outcome; consider their relevance to key groups (e.g., healthcare providers, users, and policy makers). | 15-19 | 1. MHO phenotype may not be a benign condition  2. Sex, age, and smoking status were important effect modifiers.  3. Our study is the first review and meta-analysis to indicate the elevated risk of CAC in MHO | |
| Limitations | | 25 | Discuss limitations at study and outcome level (e.g., risk of bias), and at review-level (e.g., incomplete retrieval of identified research, reporting bias). | 19-20 | Some limitations should be taken into account. | |
| **Section/topic** | **#** | | **Checklist item** | **Reported on page** | **Text excerpted from the manuscript** |  |
| Conclusions | | 26 | Provide a general interpretation of the results in the context of other evidence, and implications for future research. | 21 | 1. Our study showed a significant association between MHO and elevated risk of CAC, which in turn reflects the extent of coronary atherosclerosis. People with obesity should strive to achieve normal weight even in the absence of metabolic abnormalities.  2. Given a longer follow-up duration, we were able to detect the significant difference in CAC between MHO and MHNO  3. More studies are needed to better define metabolically healthy and benign obese phenotypes | |
| **FUNDING** | | | |  |  | |
| Funding | | 27 | Describe sources of funding for the systematic review and other support (e.g., supply of data); role of funders for the systematic review. | N/A | N/A | |

*From:*  Moher D, Liberati A, Tetzlaff J, Altman DG, The PRISMA Group (2009). Preferred Reporting Items for Systematic Reviews and Meta-Analyses: The PRISMA Statement. PLoS Med 6(7): e1000097. doi:10.1371/journal.pmed1000097

For more information, visit: **www.prisma-statement.org**.
