# Supplementary material for: Association of metabolically healthy obesity and elevated risk of coronary artery calcification: a systematic review and meta-analysis
Source: PeerJ. 2020 Mar 26;8:e8815. doi: 10.7717/peerj.8815 (PMC7103199; doi:10.7717/peerj.8815)
Supplement: Table S2 [file peerj-08-8815-s003.docx]

**Supplemental Table S2. Search strategy**

Main result

| Databases | Articles | Date | Others |
| --- | --- | --- | --- |
| PubMed | 354 | 20190419 |  |
| Cochrane CDSR | 0 | 20190419 |  |
| Cochrane CENTRAL | 80 | 20190419 |  |
| EMBASE | 1002 | 20190419 |  |
| Total | 1436 |  |  |
| endnote duplicate | 174 |  |  |
| Total without duplicated | 1262 |  |  |

Keywords

| **PICOS** |  | **MeSH terms + free text word** | **EMTree terms + free text word** |
| --- | --- | --- | --- |
| P | adult |  |  |
| I | MHO | Obesity, Metabolically Benign OR Metabolically Benign Obesity OR Metabolically Healthy Obesity OR MHO OR obesity paradox OR (obesity AND phenotypes) | metabolically benign obesity OR 'obesity paradox'/exp OR obesity paradox OR (obesity AND phenotypes) |
|  |  | Overweight OR obesity OR obese OR BODY MASS INDEX OR BMI OR fat* OR Body Weight OR Body Composition OR Anthropometry OR adiposity OR body composition OR body fat OR fatness OR body mass AND | obesity OR adiposit* OR obesitas OR overweight OR obese OR fat OR body mass OR BMI OR body mass index OR Quetelet index OR Body Weight OR Body Composition OR anthropometry OR anthropometric OR adiposity AND |
|  |  | Metabolic* AND | metaboli* AND |
|  |  | normal OR healthy OR benign OR without OR absence | normal OR healthy OR benign OR without OR absence |
| O | CAC progression | Coronary OR coronary arter* OR coronary vessel* OR coronary artery disease* OR coronary artery disease AND | Coronary OR coronary arter* OR coronary vessel* OR coronary artery disease* OR coronary artery disease AND |
|  |  | vascular calcification OR vascular calcinos* OR calcinosis OR calcinoses OR calcium OR calcification OR atheroscleros* OR atherogenesis OR arterioscleros* | vascular calcification OR vascular calcinos* OR calcinosis OR calcinoses OR calcium OR calcification OR atheroscleros* OR atherogenesis OR arterioscleros* |

Search strategy in Pubmed

| **PICO** | **Search** | **Query** | **Items found** | **Time** |
| --- | --- | --- | --- | --- |
| **PIO** | #47 | Search (((((((((Obesity, Metabolically Benign) OR Metabolically Benign Obesity) OR Metabolically Healthy Obesity) OR MHO[Title/Abstract]) OR obesity phenotypes) OR obesity phenotype*)) OR ((((((((((((((Overweight) OR obesity) OR obese[Title/Abstract]) OR Body Mass Index) OR BMI[Title/Abstract]) OR Body Weight) OR Body Composition) OR fatness[Title/Abstract]) OR body mass[Title/Abstract]) OR Anthropometry) OR adiposity)) AND ((((normal[Text Word]) OR healthy[Text Word]) OR without[Text Word]) OR absence[Text Word])) AND metaboli*[Title/Abstract]))) AND (((((((Coronary[Title/Abstract]) OR Coronary Arter*[Text Word]) OR coronary vessel*[Text Word]) OR Coronary Artery Disease) OR Coronary Artery Disease*)) AND (((((((((Vascular Calcification) OR Calcinoses [Text Word]) OR Calcinosis[Text Word]) OR Calcium[Text Word]) OR Calcification[Text Word]) OR Atheroscleros*[Title/Abstract]) OR Atherogenesis[Title/Abstract]) OR Arterioscleros*[Title/Abstract]) OR vascular calcinos*)) | 354 | 08:47:53 |
| **O** | #46 | Search ((((((Coronary[Title/Abstract]) OR Coronary Arter*[Text Word]) OR coronary vessel*[Text Word]) OR Coronary Artery Disease) OR Coronary Artery Disease*)) AND (((((((((Vascular Calcification) OR Calcinoses [Text Word]) OR Calcinosis[Text Word]) OR Calcium[Text Word]) OR Calcification[Text Word]) OR Atheroscleros*[Title/Abstract]) OR Atherogenesis[Title/Abstract]) OR Arterioscleros*[Title/Abstract]) OR vascular calcinos*) | 52128 | 08:38:51 |
|  | #45 | Search ((((((((Vascular Calcification) OR Calcinoses [Text Word]) OR Calcinosis[Text Word]) OR Calcium[Text Word]) OR Calcification[Text Word]) OR Atheroscleros*[Title/Abstract]) OR Atherogenesis[Title/Abstract]) OR Arterioscleros*[Title/Abstract]) OR vascular calcinos* | 747884 | 08:37:00 |
|  | #44 | Search vascular calcinos* | 17 | 08:35:48 |
|  | #43 | Search Arterioscleros*[Title/Abstract] | 17498 | 08:32:22 |
|  | #42 | Search Atherogenesis[Title/Abstract] | 12449 | 08:32:08 |
|  | #41 | Search Atheroscleros*[Title/Abstract] | 109791 | 08:31:53 |
|  | #40 | Search Calcification[Text Word] | 53818 | 08:30:32 |
|  | #39 | Search Calcium[Text Word] | 567971 | 08:30:10 |
|  | #38 | Search Calcinosis | 37633 | 08:29:25 |
|  | #37 | Search Calcinoses [Text Word] | 23 | 08:28:56 |
|  | #35 | Search Vascular Calcification | 10450 | 08:27:32 |
|  | #34 | Search ((((Coronary[Title/Abstract]) OR Coronary Arter*[Text Word]) OR coronary vessel*[Text Word]) OR Coronary Artery Disease) OR Coronary Artery Disease* | 413904 | 08:26:23 |
|  | #33 | Search Coronary Artery Disease* | 115836 | 08:24:56 |
|  | #32 | Search Coronary Artery Disease | 159964 | 08:24:40 |
|  | #31 | Search coronary vessel*[Text Word] | 65675 | 08:24:09 |
|  | #30 | Search Coronary Arter*[Text Word] | 235556 | 08:23:48 |
|  | #29 | Search Coronary[Title/Abstract] | 379647 | 08:22:35 |
| **I** | #28 | Search (((((((Obesity, Metabolically Benign) OR Metabolically Benign Obesity) OR Metabolically Healthy Obesity) OR MHO[Title/Abstract]) OR obesity phenotypes) OR obesity phenotype*)) OR ((((((((((((((Overweight) OR obesity) OR obese[Title/Abstract]) OR Body Mass Index) OR BMI[Title/Abstract]) OR Body Weight) OR Body Composition) OR fatness[Title/Abstract]) OR body mass[Title/Abstract]) OR Anthropometry) OR adiposity)) AND ((((normal[Text Word]) OR healthy[Text Word]) OR without[Text Word]) OR absence[Text Word])) AND metaboli*[Title/Abstract]) | 47094 | 08:00:42 |
|  | #27 | Search (((((((((((((Overweight) OR obesity) OR obese[Title/Abstract]) OR Body Mass Index) OR BMI[Title/Abstract]) OR Body Weight) OR Body Composition) OR fatness[Title/Abstract]) OR body mass[Title/Abstract]) OR Anthropometry) OR adiposity)) AND ((((normal[Text Word]) OR healthy[Text Word]) OR without[Text Word]) OR absence[Text Word])) AND metaboli*[Title/Abstract] | 36425 | 07:58:06 |
|  | #26 | Search metaboli*[Title/Abstract] | 1186770 | 07:57:32 |
|  | #25 | Search (((normal[Text Word]) OR healthy[Text Word]) OR without[Text Word]) OR absence[Text Word] | 2732359 | 07:53:29 |
|  | #24 | Search absence[Text Word] | 585428 | 07:52:47 |
|  | #23 | Search without[Text Word] | 37 | 07:52:28 |
|  | #22 | Search healthy[Text Word] | 760594 | 07:51:55 |
|  | #21 | Search normal[Text Word] | 1584341 | 07:51:45 |
|  | #20 | Search ((((((((((Overweight) OR obesity) OR obese[Title/Abstract]) OR Body Mass Index) OR BMI[Title/Abstract]) OR Body Weight) OR Body Composition) OR fatness[Title/Abstract]) OR body mass[Title/Abstract]) OR Anthropometry) OR adiposity | 1032115 | 07:50:21 |
|  | #19 | Search adiposity | 27174 | 07:47:59 |
|  | #18 | Search Anthropometry | 492643 | 07:47:41 |
|  | #17 | Search body mass[Title/Abstract] | 195196 | 07:47:25 |
|  | #16 | Search fatness[Title/Abstract] | 4016 | 07:47:07 |
|  | #15 | Search Body Composition | 84182 | 07:46:42 |
|  | #14 | Search Body Weight | 609083 | 07:46:20 |
|  | #13 | Search BMI[Title/Abstract] | 129313 | 07:45:52 |
|  | #12 | Search Body Mass Index | 222308 | 07:45:31 |
|  | #11 | Search obese[Title/Abstract] | 116348 | 07:45:07 |
|  | #9 | Search obesity | 305532 | 07:44:23 |
|  | #8 | Search Overweight | 225801 | 07:43:52 |
|  | #7 | Search (((((Obesity, Metabolically Benign) OR Metabolically Benign Obesity) OR Metabolically Healthy Obesity) OR MHO[Title/Abstract]) OR obesity phenotypes) OR obesity phenotype* | 12757 | 07:42:59 |
|  | #6 | Search obesity phenotype* | 566 | 07:40:43 |
|  | #5 | Search obesity phenotypes | 12010 | 07:40:23 |
|  | #4 | Search MHO[Title/Abstract] | 526 | 07:39:29 |
|  | #3 | Search Metabolically Healthy Obesity | 986 | 07:37:23 |
|  | #1 | Search Obesity, Metabolically Benign | 208 | 07:35:56 |
|  | #2 | Search Metabolically Benign Obesity | 208 | 07:35:51 |

Search strategy in Embase

|  | **No.** | **Query** | **Results** |
| --- | --- | --- | --- |
| **PIO** | #46 | ((('metabolically benign obesity'/exp OR 'metabolically benign obesity') OR mho:ti,ab OR 'obesity paradox'/exp OR (obesity AND phenotypes) OR 'metabolically healthy obesity'/exp) OR ((overweight:ti,ab,kw OR obesity:ti,ab,kw OR adiposit*:ti,ab,kw OR fat:ti,ab,kw OR 'body mass'/exp OR bmi:ti,ab,kw OR 'body mass index':ti,ab,kw OR 'body weight'/exp OR 'body composition'/exp OR fat:ti,ab,kw OR 'anthropometry'/exp) AND ((normal:ti,ab,kw OR healthy:ti,ab,kw OR benign:ti,ab,kw OR without:ti,ab,kw OR absence:ti,ab,kw) AND metaboli*:ti,ab))) AND ((coronary:ti,ab OR 'coronary arter*':ti,ab,kw OR 'coronary vessel*':ti,ab,kw OR 'coronary artery disease*' OR 'coronary artery disease'/exp) AND ('vascular calcification'/exp OR 'vascular calcinos*' OR calcinosis:ti,ab,kw OR calcinoses:ti,ab,kw OR calcium:ti,ab,kw OR calcification:ti,ab,kw OR atheroscleros*:ti,ab OR atherogenesis:ti,ab OR arterioscleros*:ti,ab)) | 1002 |
| **O** | #45 | (coronary:ti,ab OR 'coronary arter*':ti,ab,kw OR 'coronary vessel*':ti,ab,kw OR 'coronary artery disease*' OR 'coronary artery disease'/exp) AND ('vascular calcification'/exp OR 'vascular calcinos*' OR calcinosis:ti,ab,kw OR calcinoses:ti,ab,kw OR calcium:ti,ab,kw OR calcification:ti,ab,kw OR atheroscleros*:ti,ab OR atherogenesis:ti,ab OR arterioscleros*:ti,ab) | 72533 |
|  | #44 | 'vascular calcification'/exp OR 'vascular calcinos*' OR calcinosis:ti,ab,kw OR calcinoses:ti,ab,kw OR calcium:ti,ab,kw OR calcification:ti,ab,kw OR atheroscleros*:ti,ab OR atherogenesis:ti,ab OR arterioscleros*:ti,ab | 671306 |
|  | #43 | arterioscleros*:ti,ab | 14621 |
|  | #42 | atherogenesis:ti,ab | 15950 |
|  | #41 | atheroscleros*:ti,ab | 147497 |
|  | #40 | calcification:ti,ab,kw | 58666 |
|  | #39 | calcium:ti,ab,kw | 459470 |
|  | #38 | calcinoses:ti,ab,kw | 32 |
|  | #37 | calcinosis:ti,ab,kw | 4985 |
|  | #36 | 'vascular calcinos*' | 21 |
|  | #35 | 'vascular calcification'/exp | 18202 |
|  | #34 | coronary:ti,ab OR 'coronary arter*':ti,ab,kw OR 'coronary vessel*':ti,ab,kw OR 'coronary artery disease*' OR 'coronary artery disease'/exp | 623062 |
|  | #33 | 'coronary artery disease'/exp | 315740 |
|  | #32 | 'coronary artery disease*' | 238264 |
|  | #31 | 'coronary vessel*':ti,ab,kw | 8742 |
|  | #30 | 'coronary arter*':ti,ab,kw | 284418 |
|  | #29 | coronary:ti,ab | 532402 |
| **I** | #28 | (('metabolically benign obesity'/exp OR 'metabolically benign obesity') OR mho:ti,ab OR 'obesity paradox'/exp OR (obesity AND phenotypes) OR 'metabolically healthy obesity'/exp) OR ((overweight:ti,ab,kw OR obesity:ti,ab,kw OR adiposit*:ti,ab,kw OR fat:ti,ab,kw OR 'body mass'/exp OR bmi:ti,ab,kw OR 'body mass index':ti,ab,kw OR 'body weight'/exp OR 'body composition'/exp OR fat:ti,ab,kw OR 'anthropometry'/exp) AND ((normal:ti,ab,kw OR healthy:ti,ab,kw OR benign:ti,ab,kw OR without:ti,ab,kw OR absence:ti,ab,kw) AND metaboli*:ti,ab)) | 84687 |
|  | #27 | (overweight:ti,ab,kw OR obesity:ti,ab,kw OR adiposit*:ti,ab,kw OR fat:ti,ab,kw OR 'body mass'/exp OR bmi:ti,ab,kw OR 'body mass index':ti,ab,kw OR 'body weight'/exp OR 'body composition'/exp OR fat:ti,ab,kw OR 'anthropometry'/exp) AND ((normal:ti,ab,kw OR healthy:ti,ab,kw OR benign:ti,ab,kw OR without:ti,ab,kw OR absence:ti,ab,kw) AND metaboli*:ti,ab) | 79332 |
|  | #26 | (normal:ti,ab,kw OR healthy:ti,ab,kw OR benign:ti,ab,kw OR without:ti,ab,kw OR absence:ti,ab,kw) AND metaboli*:ti,ab | 361478 |
|  | #25 | metaboli*:ti,ab | 1396764 |
|  | #24 | normal:ti,ab,kw OR healthy:ti,ab,kw OR benign:ti,ab,kw OR without:ti,ab,kw OR absence:ti,ab,kw | 5699264 |
|  | #23 | absence:ti,ab,kw | 737167 |
|  | #22 | without:ti,ab,kw | 2355052 |
|  | #21 | benign:ti,ab,kw | 288111 |
|  | #20 | healthy:ti,ab,kw | 1046940 |
|  | #19 | normal:ti,ab,kw | 2105003 |
|  | #18 | overweight:ti,ab,kw OR obesity:ti,ab,kw OR adiposit*:ti,ab,kw OR fat:ti,ab,kw OR 'body mass'/exp OR bmi:ti,ab,kw OR 'body mass index':ti,ab,kw OR 'body weight'/exp OR 'body composition'/exp OR fat:ti,ab,kw OR 'anthropometry'/exp | 1462916 |
|  | #17 | 'anthropometry'/exp | 79019 |
|  | #16 | fat:ti,ab,kw | 323726 |
|  | #15 | 'body composition'/exp | 94136 |
|  | #14 | 'body weight'/exp | 633121 |
|  | #13 | 'body mass index':ti,ab,kw | 239448 |
|  | #12 | bmi:ti,ab,kw | 274573 |
|  | #11 | 'body mass'/exp | 376546 |
|  | #10 | fat:ti,ab,kw | 323726 |
|  | #9 | adiposit*:ti,ab,kw | 32385 |
|  | #8 | obesity:ti,ab,kw | 341322 |
|  | #7 | overweight:ti,ab,kw | 94303 |
|  | #6 | ('metabolically benign obesity'/exp OR 'metabolically benign obesity') OR mho:ti,ab OR 'obesity paradox'/exp OR (obesity AND phenotypes) OR 'metabolically healthy obesity'/exp | 6857 |
|  | #5 | 'metabolically healthy obesity'/exp | 82 |
|  | #4 | obesity AND phenotypes | 6125 |
|  | #3 | 'obesity paradox'/exp | 28 |
|  | #2 | mho:ti,ab | 811 |
|  | #1 | 'metabolically benign obesity'/exp OR 'metabolically benign obesity' | 53 |

Search strategy in Cochrane Library

| **PICO** | **Search** | **Query** |
| --- | --- | --- |
|  | #1 | MeSH descriptor: [Obesity, Metabolically Benign] explode all trees |
|  | #2 | ((Metabolically Healthy Obesity):ti,ab,kw OR (Metabolically Benign Obesity):ti,ab,kw OR (MHO):ti,ab,kw OR (obesity paradox):ti,ab,kw OR obesity paradox OR obesity phenotypes OR obesity phenotype*):ti,ab,kw |
|  | #3 | #1 OR #2 |
|  | #4 | (Overweight OR obesity OR obese OR Body Mass Index OR BMI OR Body Weight OR Body Composition OR fatness OR body mass OR Anthropometry OR adiposity):ti,ab,kw (Word variations have been searched) |
|  | #5 | (metaboli*):ti,ab,kw (Word variations have been searched) |
|  | #6 | (normal OR healthy OR benign OR without OR absence):ti,ab,kw (Word variations have been searched) |
|  | #7 | #4 AND #5 AND #6 |
| **I** | #8 | #3 OR #7 |
|  | #9 | MeSH descriptor: [Coronary Artery Disease] explode all trees |
|  | #10 | MeSH descriptor: [Coronary Vessels] explode all trees |
|  | #11 | (Coronary Artery Disease* OR coronary vessel* OR Coronary Arter* OR Coronary):ti,ab,kw (Word variations have been searched) |
|  | #12 | #9 OR #10 OR #11 |
|  | #13 | MeSH descriptor: [Vascular Calcification] explode all trees |
|  | #14 | (Calcinoses OR Calcium OR Calcification OR Atheroscleros* OR Atherogenesis OR Arterioscleros* OR vascular calcinos*):ti,ab,kw (Word variations have been searched) |
|  | #15 | MeSH descriptor: [Calcinosis] explode all trees |
|  | #16 | #13 OR #14 OR #15 |
| **O** | #17 | #12 AND #16 |
| **PIO** | #18 | #8 AND #17 |
