# Supplementary material for: Association of metabolically healthy obesity and elevated risk of coronary artery calcification: a systematic review and meta-analysis
Source: PeerJ. 2020 Mar 26;8:e8815. doi: 10.7717/peerj.8815 (PMC7103199; doi:10.7717/peerj.8815)
Supplement: Table S3-1 — Each star represents if individual criterion within the subsection was fulfilled. [file peerj-08-8815-s004.docx]

**Supplemental Table S3-1. Newcastle-Ottawa scale for assessment of quality of included studies – Cohort studies**

(each star represents if individual criterion within the subsection was fulfilled)

| Study | Selection | | | | Comparability | | Outcome | | | | **Total quality**  **score** | |
| --- | --- | --- | --- | --- | --- | --- | --- | --- | --- | --- | --- | --- |
| Quality assessment criteria | Representativeness of  exposed  cohort | Selection of non−exposed cohort | Ascertainment  of exposure | Demonstration that outcome  of interest was not present at start of study | Adjust for the most important risk factors | Adjust for other risk factors | | Assessment of outcome | Follow−up  length | Loss to follow−up rate | |  |
| Acceptable (★) | Representative of general adult population in community (age/sex/being at risk of disease) | Drawn from the same community as exposed cohort | Secure records, Structured interview | Yes, or excluded when analysis | Yes, at least for age and sex | Yes, and smoking must be included | | Independent blind assessment, record linkage | Follow−up >1 years | Follow−up completed, or small subjects lost(<20%), or lost subjects unlikely to introduce bias* | |  |
| Yoon, 2017 | ★ | ★ | ★ | ★ | ★ | − | | ★ | ★ | ★ | | **8** |
| Kang, 2017 | ★ | ★ | ★ | ★ | ★ | ★ | | ★ | ★ | ★ | | **9** |
| Kowall, 2018 | ★ | ★ | ★ | ★ | ★ | ★ | | ★ | ★ | ★ | | **9** |
