# Supplementary material for: Association of metabolically healthy obesity and elevated risk of coronary artery calcification: a systematic review and meta-analysis
Source: PeerJ. 2020 Mar 26;8:e8815. doi: 10.7717/peerj.8815 (PMC7103199; doi:10.7717/peerj.8815)
Supplement: Table S3-2 — Each star represents if individual criterion within the subsection was fulfilled. [file peerj-08-8815-s005.docx]

**Supplemental Table S3-2. Newcastle-Ottawa scale for assessment of quality of included studies – Cross-sectional studies**

(each star represents if individual criterion within the subsection was fulfilled)

| Study | Selection | | | | Comparability | | Outcome | | **Total quality**  **score** |
| --- | --- | --- | --- | --- | --- | --- | --- | --- | --- |
| Quality assessment criteria | Representativeness of the sample | Sample size | Non-respondents | Ascertainment of the exposure (risk factor) | Adjust for the most important risk factors | Adjust for other risk factors | Assessment of outcome | Statistical test |  |
| Acceptable (★) | Representative of general adult population in community (age/sex/being at risk of disease) | Justified and satisfactory (including sample size calculation) | Proportion of target sample attains pre-specified target or non-respondent characteristics recorded | Validated  measurement tool (two stars)  Non-validated  measurementtool, but the tool is  described.  (one star) | Yes, at least for age and sex | Yes, and smoking must be included | CAC assessed by CT (two stars),  by other method (one star) | The statistical test clearly  described/ confidence intervals and p value included |  |
| Khan, 2011 | ★ | −­ | ★ | ★★ | ★ | ★ | ★★ | ★ | **9** |
| Rhee, 2013 | ★ | − | − | ★★ | ★ | ★ | ★★ | − | **7** |
| Sung, 2014 | ★ | − | − | ★★ | ★ | ★ | ★★ | ★ | **8** |
| Chang, 2014 | ★ | − | − | ★★ | ★ | ★ | ★★ | − | **7** |
| Jung C.H., 2014 | ★ | − | − | ★★ | ★ | ★ | ★★ | ★ | **8** |
| Echouffo-Tcheugui, 2019 | ★ | − | − | ★★ | ★ | ★ | ★★ | − | **7** |
