# Supplementary figures and images for: Association of metabolically healthy obesity and elevated risk of coronary artery calcification: a systematic review and meta-analysis
Source: PeerJ. 2020 Mar 26;8:e8815. doi: 10.7717/peerj.8815 (PMC7103199; doi:10.7717/peerj.8815)

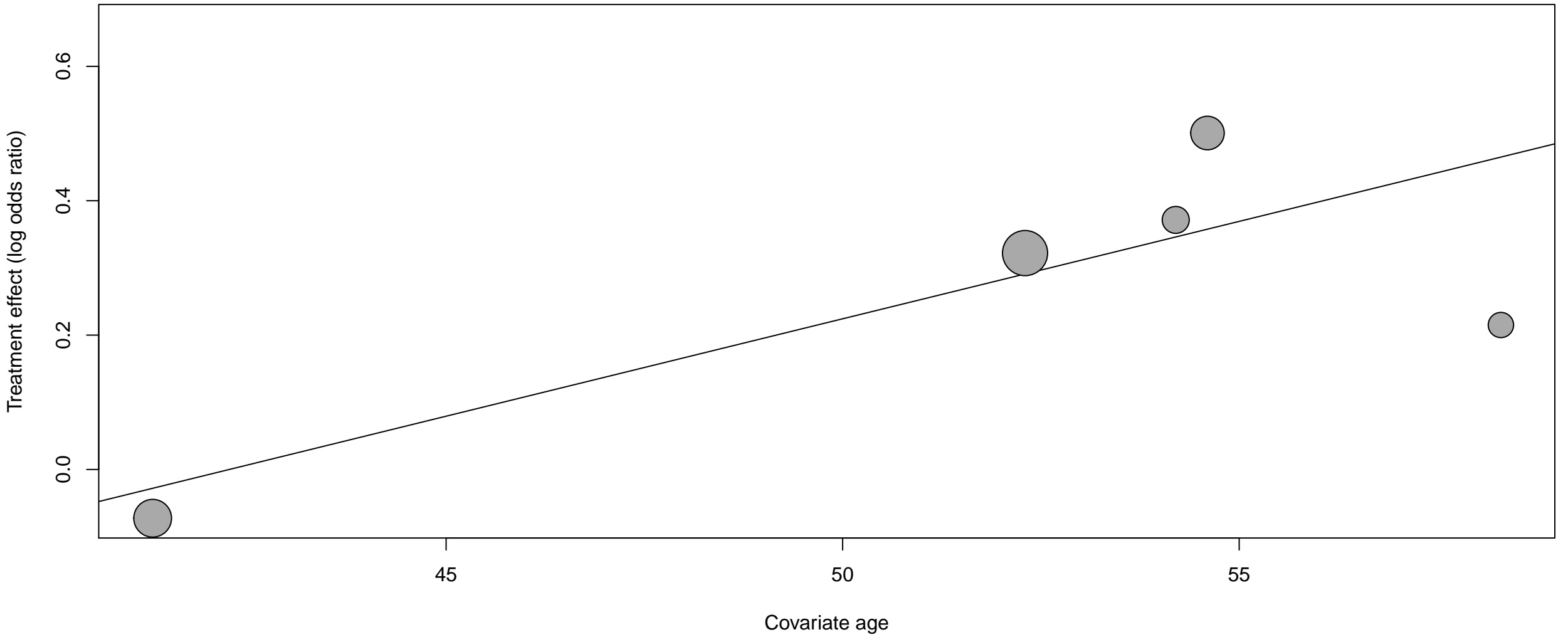

Supplement: Figure S1 — Each bubble represented a study and bubble size represented the sample size of the study. The regression line showed a non-significant trend of increasing risk with age increased. (p = 0.08) [file peerj-08-8815-s006.pdf]

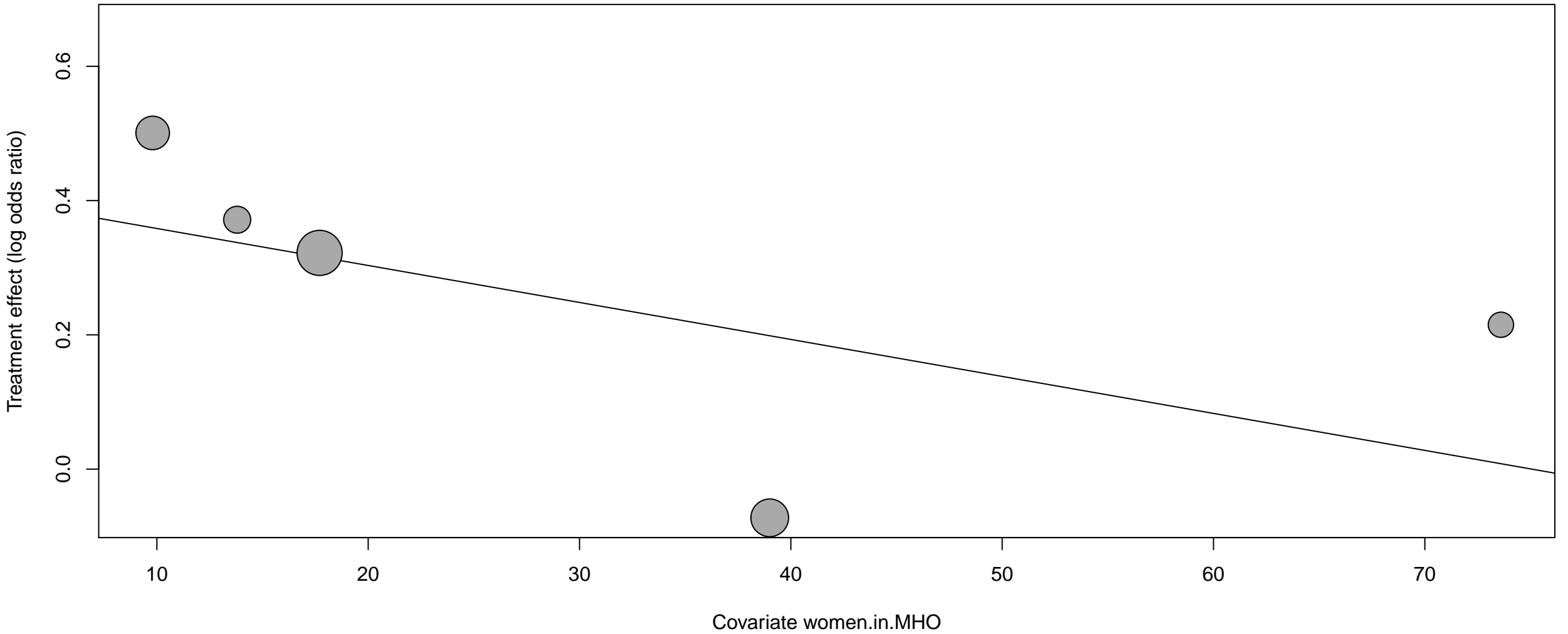

Supplement: Figure S2 — Each bubble represented a study and bubble size represented the sample size of the study. The regression line showed a non-significant trend of declining risk with women proportion increase. (p = 0.31) [file peerj-08-8815-s007.pdf]

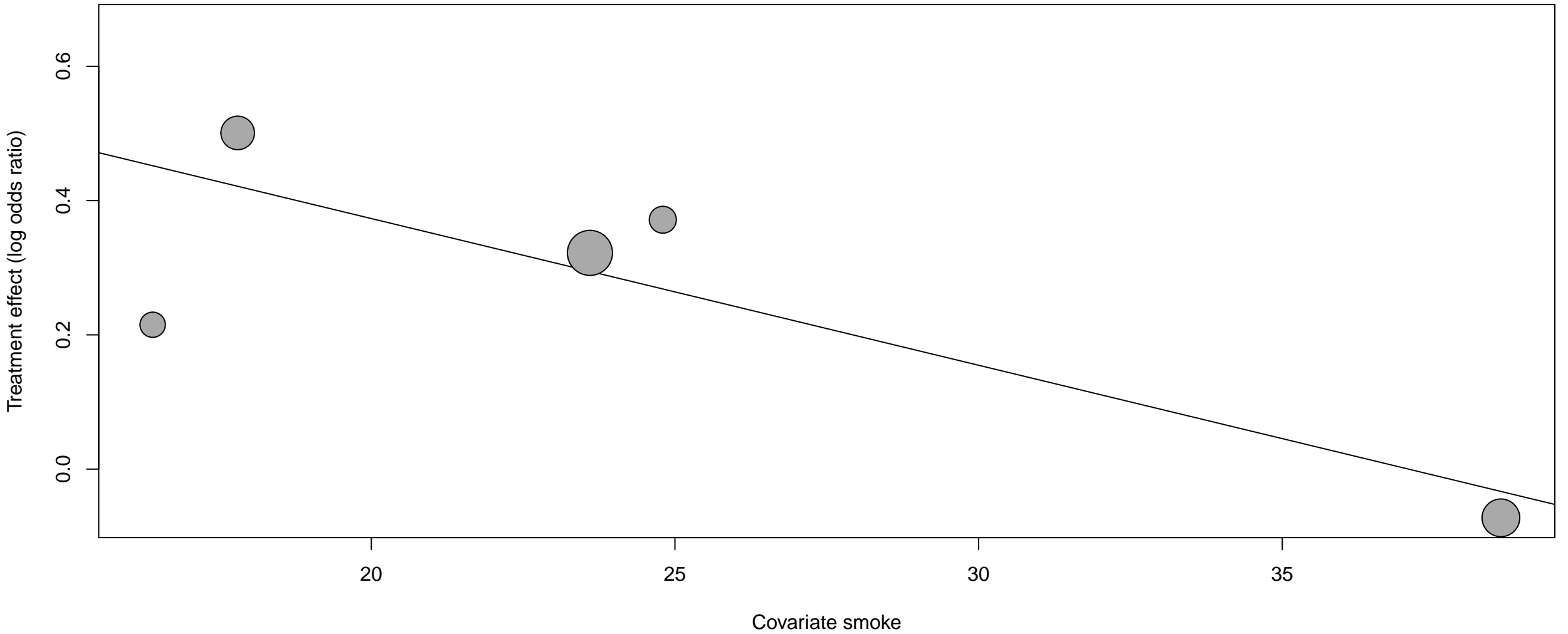

Supplement: Figure S3 — Each bubble represented a study and bubble size represented the sample size of the study. The regression line showed borderline significant trend of decreased risk with smoke proportion increased. (p = 0.058) [file peerj-08-8815-s008.pdf]

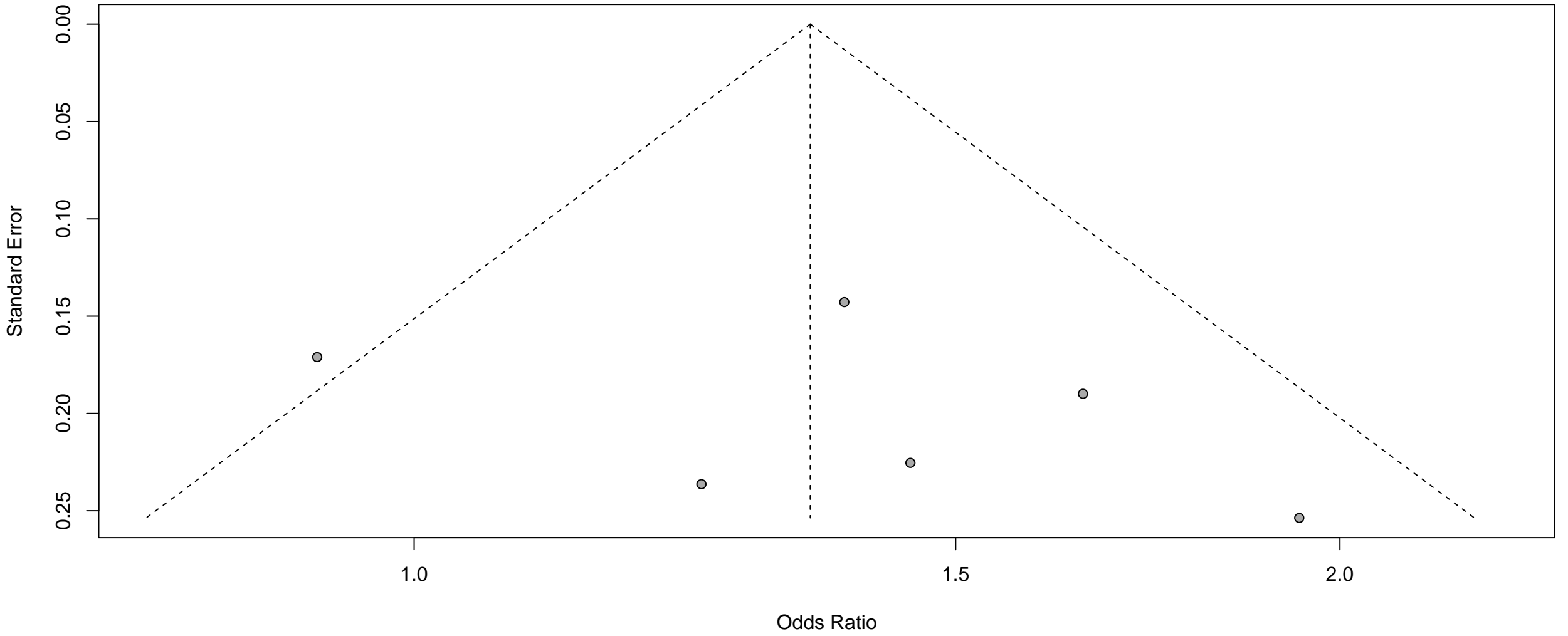

Supplement: Figure S4 [file peerj-08-8815-s009.pdf]
