# Supplementary material for: Association of metabolically healthy obesity and elevated risk of coronary artery calcification: a systematic review and meta-analysis
Source: PeerJ. 2020 Mar 26;8:e8815. doi: 10.7717/peerj.8815 (PMC7103199; doi:10.7717/peerj.8815)
Supplement: Supplemental Information 1 [file peerj-08-8815-s010.docx]

**Statement of Rationale and Contribution of the work.**

**1.The rationale for conducting the meta-analysis**

Some studies report that MHO individuals show similar risk of cardiovascular disease (CVD) compared with metabolically healthy non-obese (MHNO) individuals, but the results are conflicting. No uniform definition of MHO was established during these studies. Coronary artery calcium (CAC) reflects the extent of coronary atherosclerosis and is a useful tool to predict future risk of CVD. Our meta-analysis was to investigate whether MHO is associated with elevated risk of CAC.

**2. The contribution that the meta-analysis makes to knowledge in light of previously published related reports, including other meta-analyses and systematic reviews.**

Our study is the first systematic review and meta-analysis to show that MHO phenotype increases the risk of CAC progression, which reflects the extent of coronary atherosclerosis and quite possibly indicates increased future risk of CVD. People with obesity still should strive to achieve normal weight even in the absence of metabolic abnormalities.
